# Supplementary figures and images for: Genome-wide functional analysis reveals key roles for kinesins in the mammalian and mosquito stages of the malaria parasite life cycle
Source: PLoS Biol. 2022 Jul 28;20(7):e3001704. doi: 10.1371/journal.pbio.3001704 (PMC9333250; doi:10.1371/journal.pbio.3001704)

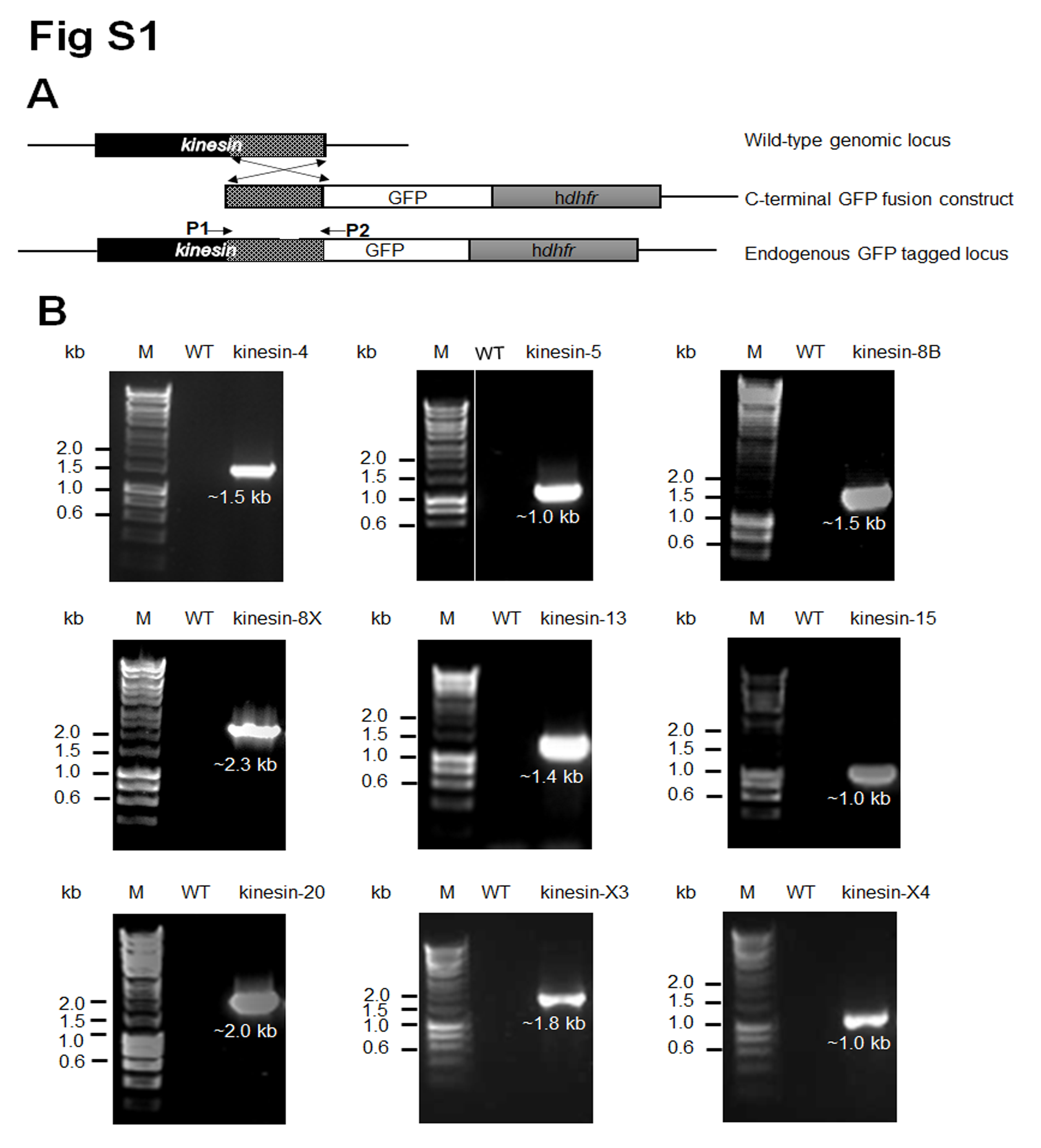

Supplement: S1 Fig — (A) Schematic representation of the endogenous kinesin locus, the GFP-tagging construct, and the recombined kinesin locus following single homologous recombination. Arrows 1 (P1) and 3 (P3) indicate the position of PCR primers used to confirm successful integration of the construct. (B) Diagnostic PCR of kinesin and WT parasites using primers: integration primer (P1) and ol492 (P2). The bands of expected size for amplified DNA fragments are indicated for each tagged line. (TIF) [file pbio.3001704.s001.tif]

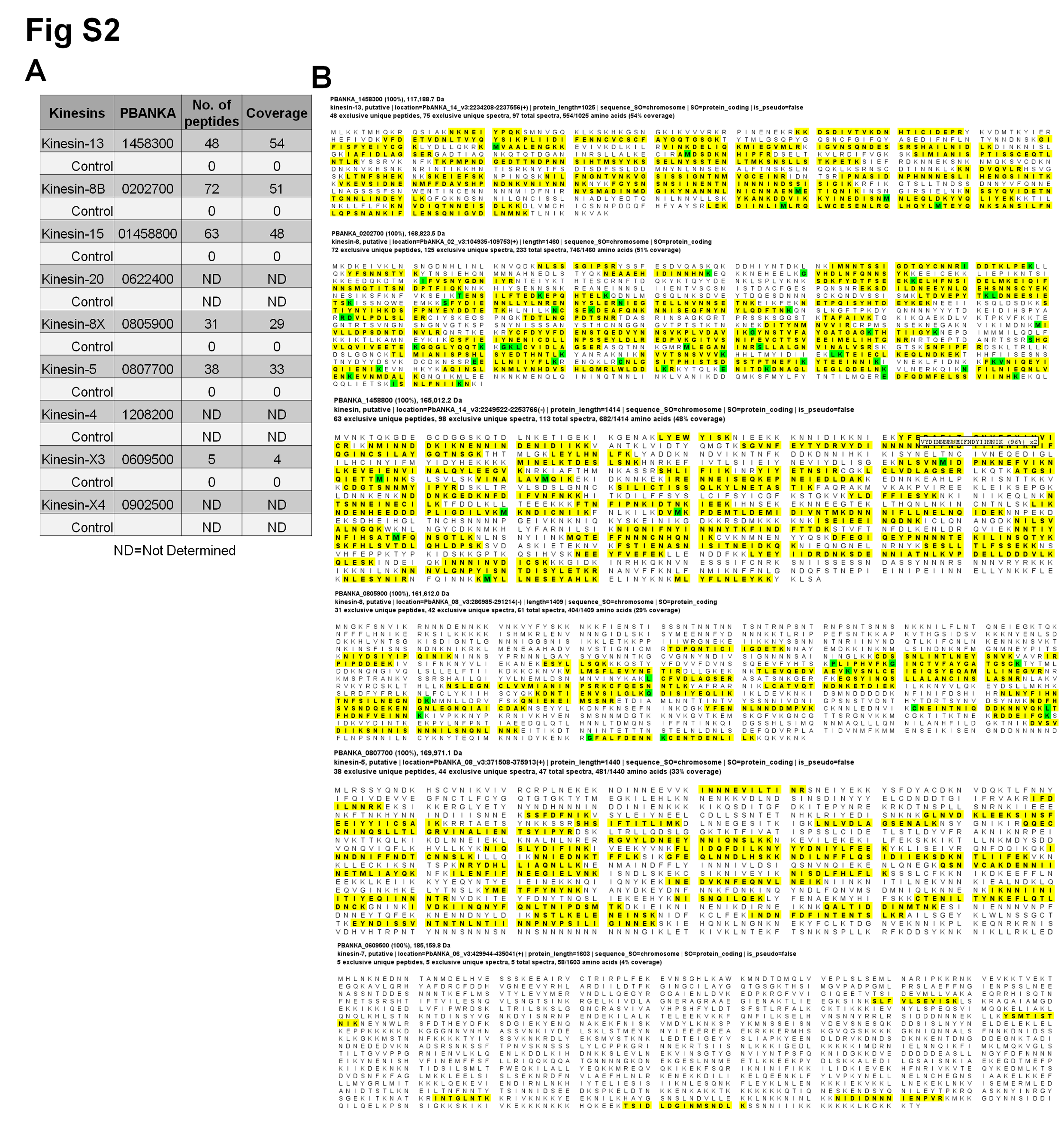

Supplement: S2 Fig — (A) The number of peptides after immunoprecipitation using GFP-trap beads and their coverage to full length kinesins. (B) Locations of peptides in full-length kinesin proteins. (TIF) [file pbio.3001704.s002.tif]

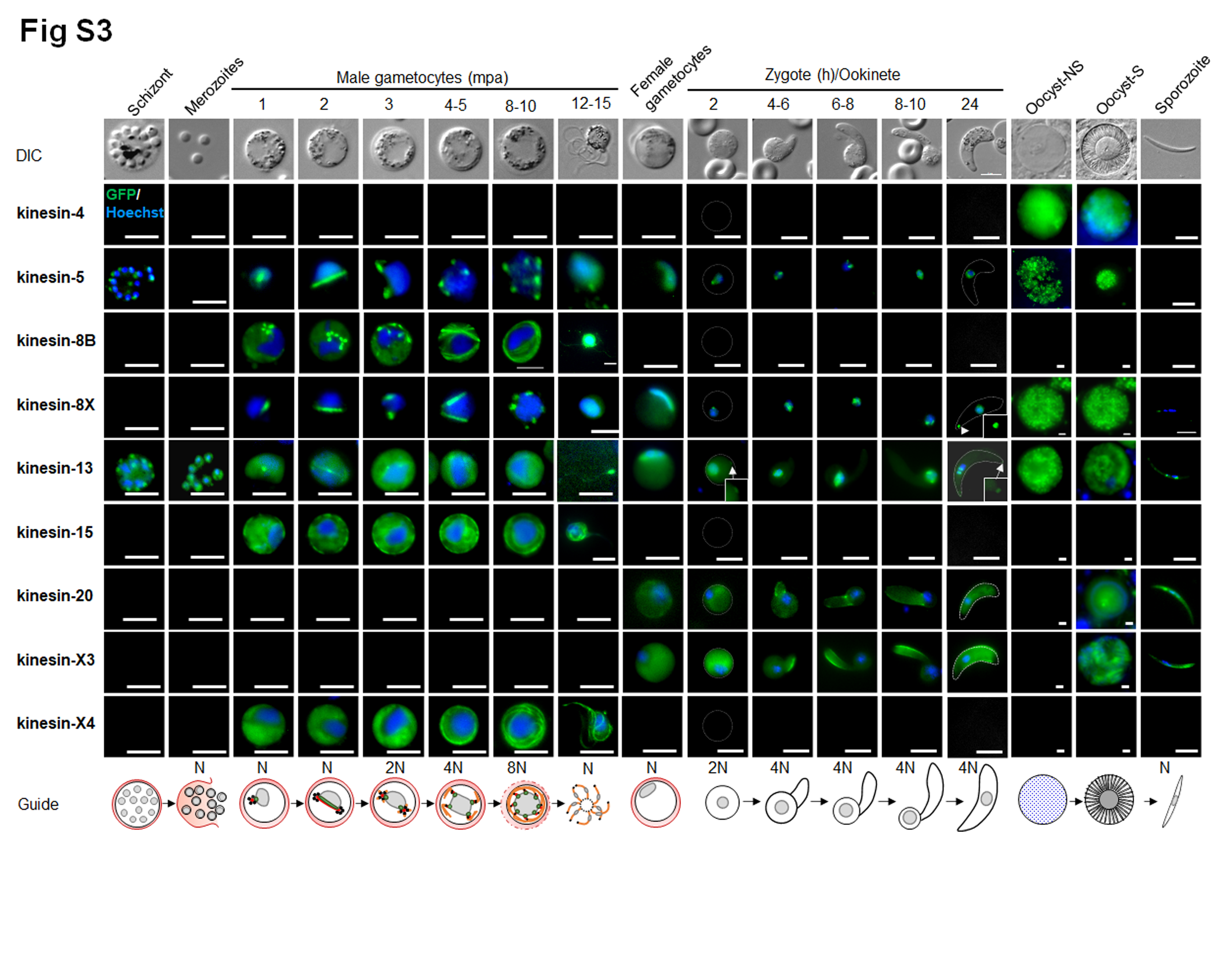

Supplement: S3 Fig — Live cell imaging showing subcellular locations of 9 kinesin-GFP proteins (green) during various stages of the P. berghei life cycle. DNA is stained with Hoechst dye (blue). Arrowhead indicates basal end and arrow indicates apical end of the ookinete. Scale bar = 5 μm. mpa, min post-activation; Oocyst-NS, nonsporulating oocyst; Oocyst-S, sporulating oocyst. (TIF) [file pbio.3001704.s003.tif]

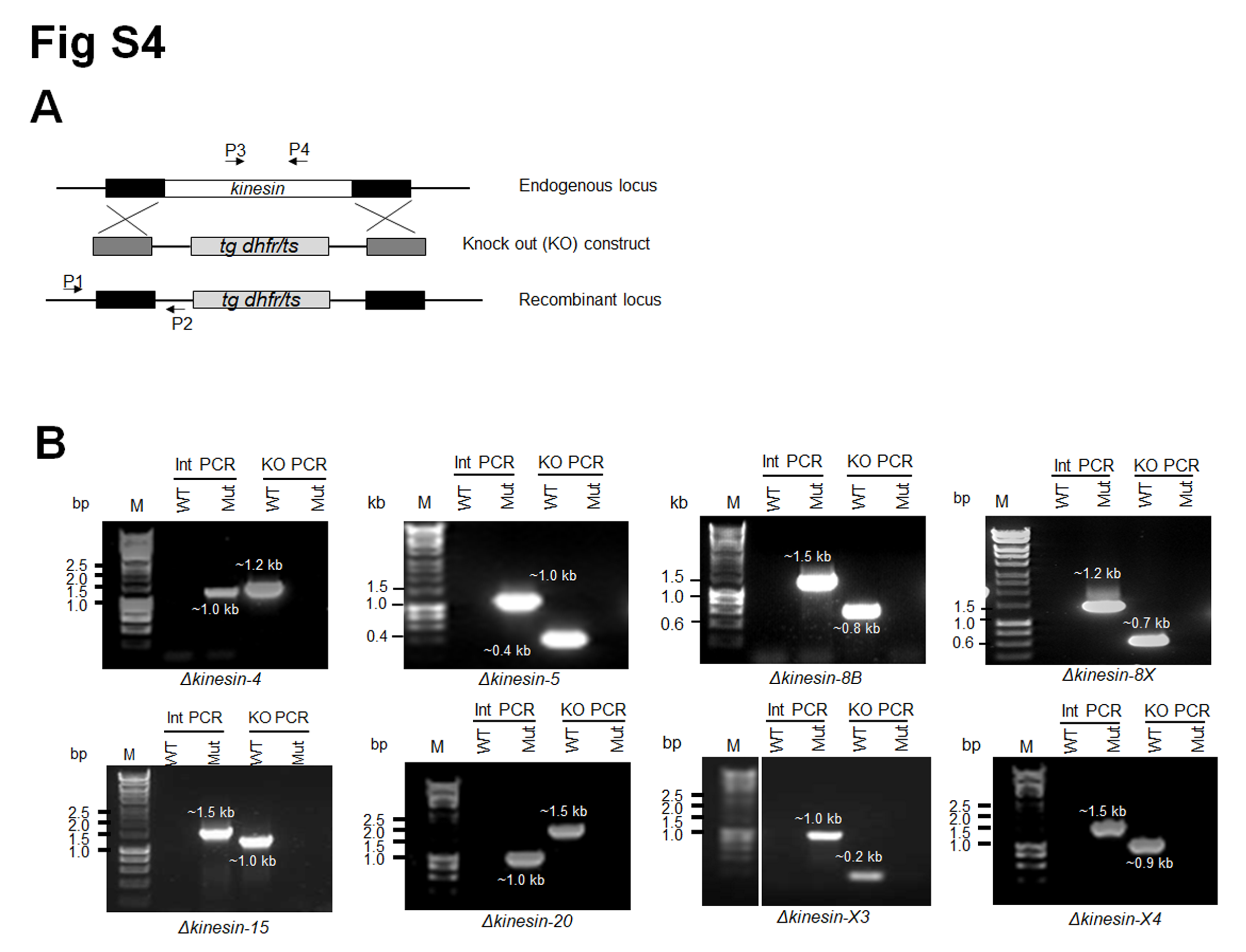

Supplement: S4 Fig — (A) Schematic representation of the endogenous kinesin locus, the targeting knockout construct, and the recombined kinesin locus following double homologous crossover recombination. Arrows 1 and 2 indicate PCR primers used to confirm successful integration in the kinesin locus following recombination, and arrows 3 and 4 indicate PCR primers used to show deletion of the kinesin gene. (B) Integration PCR of the kinesin locus in WTGFP (WT) and knockout (Mut) parasites using primers: integration primer and ol248. Integration of the targeting construct gives expected size band for each gene. (TIF) [file pbio.3001704.s004.tif]

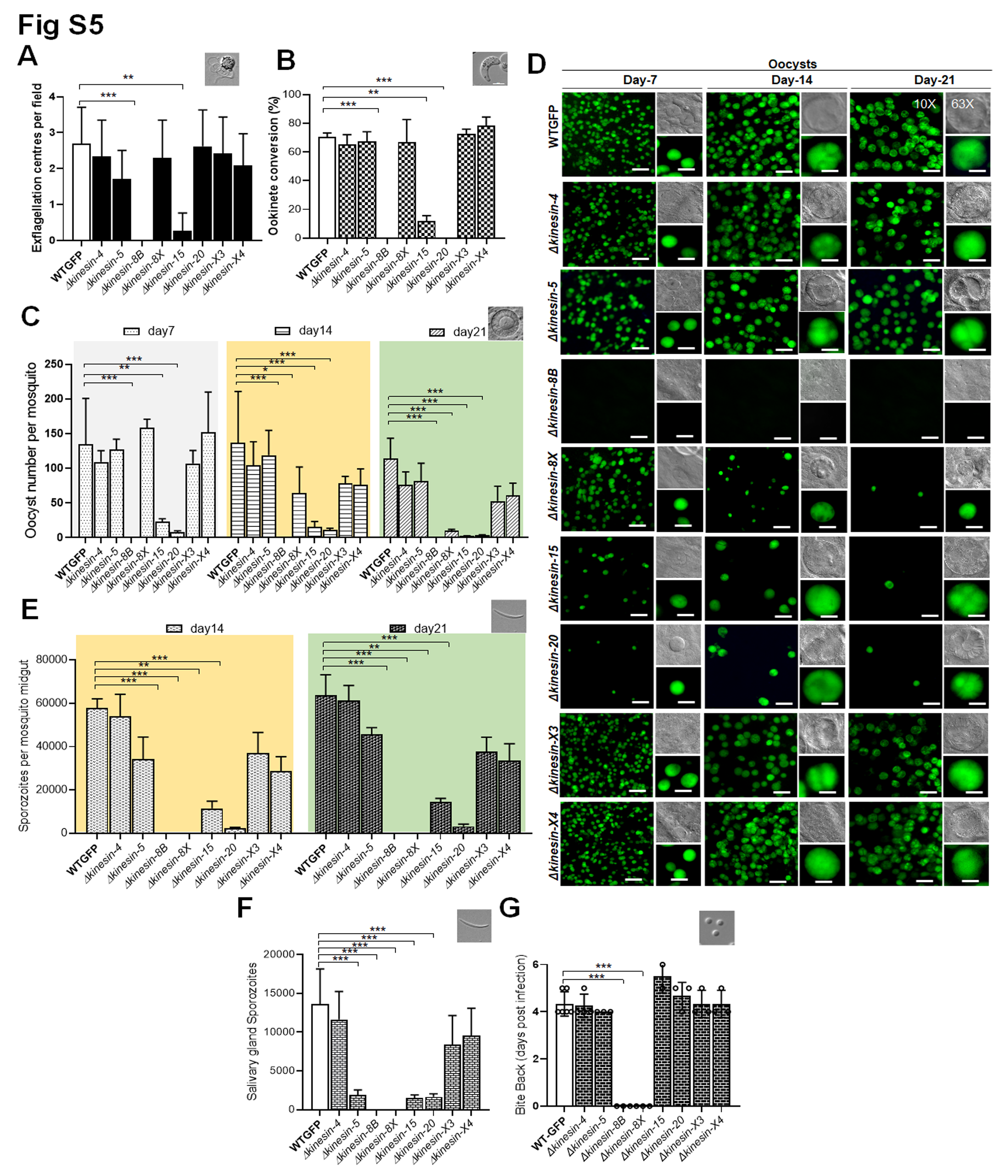

Supplement: S5 Fig — (A) Male gametogenesis in kinesin gene-deletion lines in comparison with WTGFP parasite, measured as the number of exflagellation centres per field; more than 20 fields were counted for each line. Mean ± SEM. n = 3 independent experiments. (B) Percentage ookinete conversion comparing knockout and WTGFP parasites. Ookinetes were identified using 13.1 antibody for surface marker (P28, red) and defined as those cells that differentiated successfully into elongated “banana-shaped” ookinetes. Mean ± SEM. n = 5 independent experiments. (C) Total number of GFP-positive oocysts per mosquito midgut at 7, 14, and 21 dpi for knockout and WTGFP parasites; at least 10 mosquito midguts were counted for each line. Mean ± SEM. n = 3 independent experiments. (D) Representative mosquito midguts at 10× and 63× magnification showing oocysts of kinesin knockout and WTGFP lines at 7, 14, and 21 dpi. Scale bar = 50 μm (10×), 20 μm (63×). (E) Total number of sporozoites in oocysts of kinesin knockout and WTGFP parasites at 14 and 21 dpi. Mean ± SEM. n = 3 independent experiments. (F) Total number of sporozoites in salivary glands of kinesin knockout and WT-GFP parasites. Mean ± SEM. n = 3 independent experiments. (G) Mosquito bite back experiments showing no transmission of Δkinesin-8B and Δkinesin-8X parasites, while other kinesin knockout and WTGFP parasites show successful transmission from mosquito to mice. Mean ± SEM. n = 3 independent experiments. *p ≤ 0.05, **p ≤ 0.01, and ***p ≤ 0.001. Underlying data are provided in the Supporting information as S3 Data. dpi, days post-infection. (TIF) [file pbio.3001704.s005.tif]

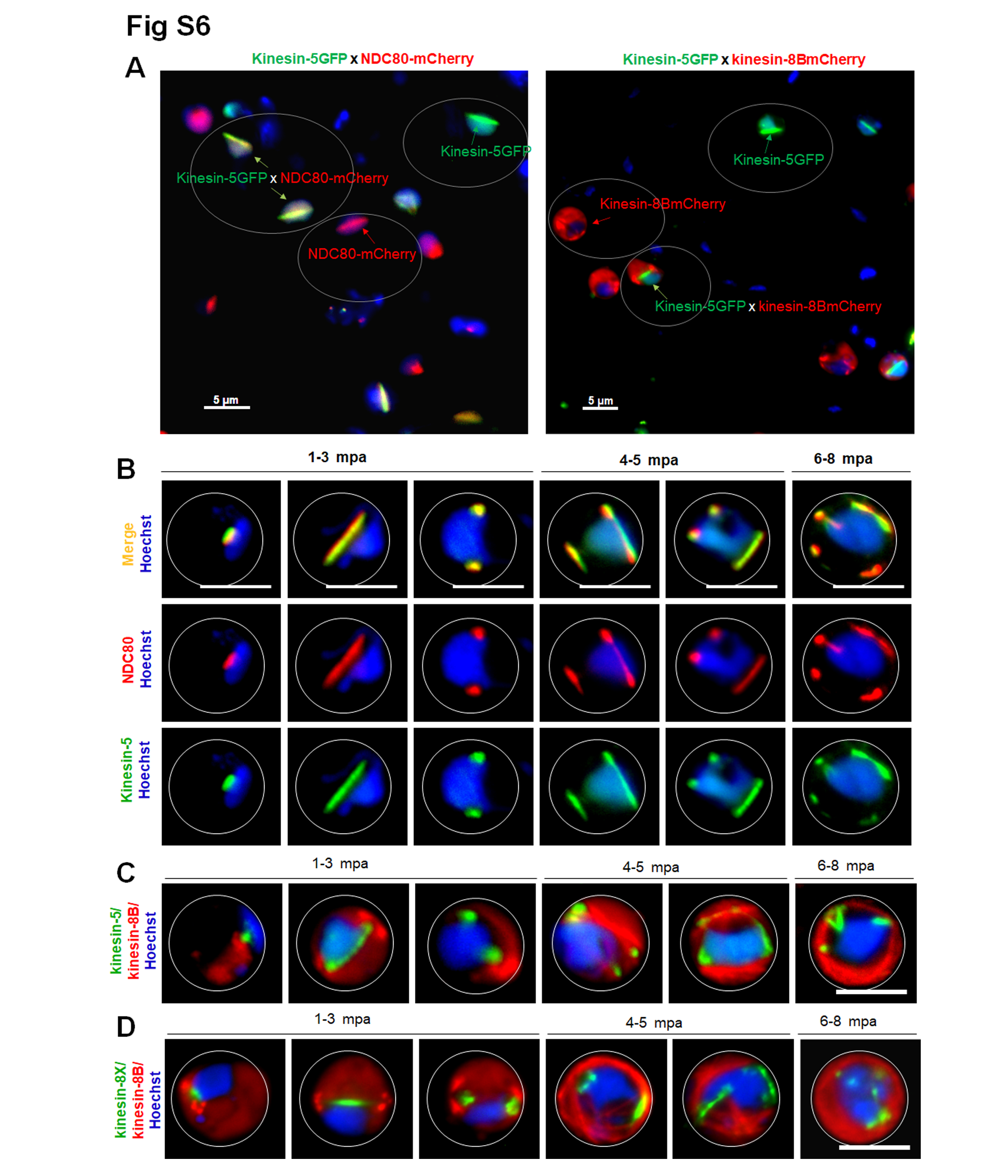

Supplement: S6 Fig — (A) Live cell images of gametocytes showing dual lines expressing either kinesin-5GFP or NDC80-mCherry/kinesin-8BmCherry or both in the same gametocyte. (B) Live cell imaging showing the temporal dynamics of kinesin-5GFP (green) along with kinetochore marker NDC80Cherry (red) during male gametogenesis. DNA is stained with Hoechst dye (blue); scale bar = 5 μm. (C). The location of kinesin-5GFP (green) in relation to the axoneme marker, kinesin-8BCherry (red) during male gamete formation. (D). The location of kinesin-8XGFP (green) in relation to the axoneme marker, kinesin-8BCherry (red) during male gamete formation. The nuclear location of kinesin-5 and kinesin-8X contrasts with the cytoplasmic location of kinesin-8B during chromosome replication and segregation, indicating that kinesin-5 and kinesin-8X are associated with the mitotic spindle. DNA is stained with Hoechst dye (blue). Scale bar = 5 μm. mpa, min post-activation; (TIF) [file pbio.3001704.s006.tif]

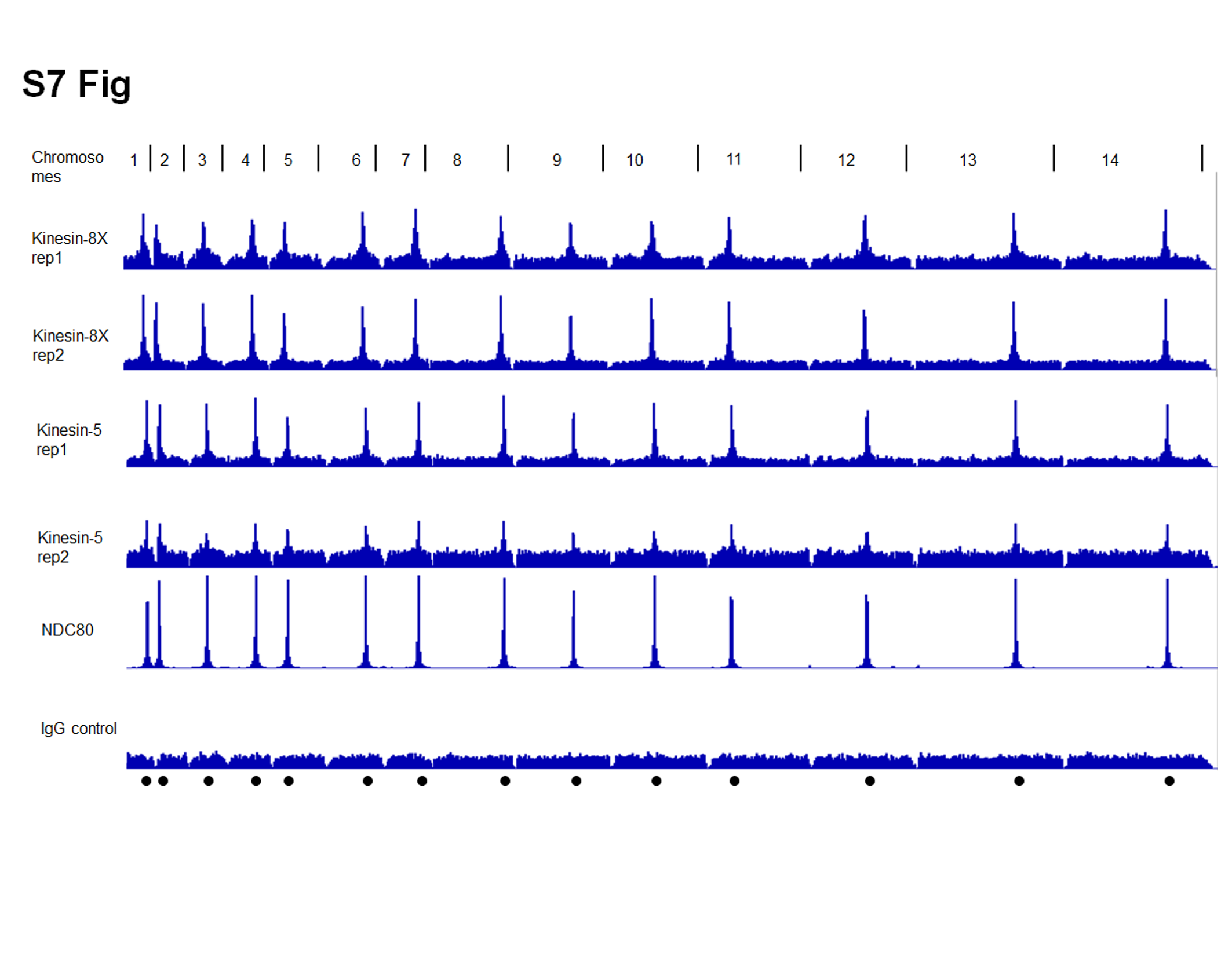

Supplement: S7 Fig — Centromeric localization confirmed by ChIP-seq analysis of kinesin-8XGFP and kinesin-5GFP profiles for all 14 chromosomes in gametocyte stage. Signals are plotted on a normalised RPM basis. Lines on top are division points between chromosomes, and circles on the bottom indicate locations of centromeres. NDC80-GFP was used as a positive control, and IgG was used as a negative control. IgG, immunoglobulin G; RPM, read per million. (TIF) [file pbio.3001704.s007.tif]

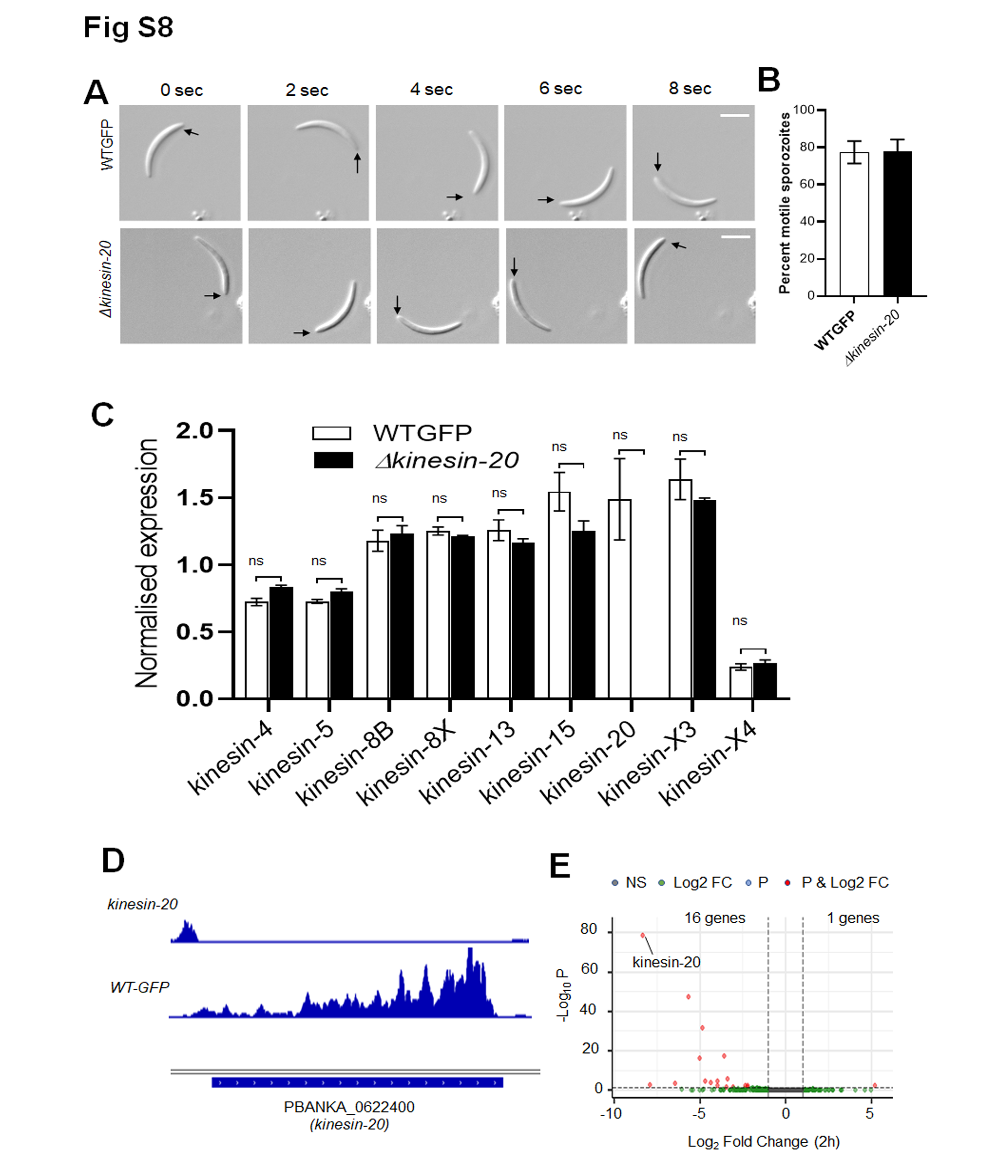

Supplement: S8 Fig — (A) Representative frames from time-lapse videos showing motile sporozoites of WTGFP and Δkinesin-20 lines. Black arrow indicates the apical end of the sporozoites. Scale bar = 5 μm. (B) Sporozoite motility for WTGFP and Δkinesin-20 lines. More than 20 sporozoites were examined for each line. Mean ± SEM. n = 3 independent experiments. (C) qRT-PCR analysis of transcripts for other kinesin genes in Δkinesin-20 and WTGFP parasites. Mean ± SEM. n = 3 independent experiments. (D) RNA-seq analysis showing no transcript in Δkinesin-20 parasites. (E) Volcano plot showing differentially regulated genes in Δkinesin-20 gametocytes activated for 2 h. Underlying data are provided in the Supporting information as S4 Data. ns, not significant; qRT-PCR, quantitative real-time PCR. (TIF) [file pbio.3001704.s008.tif]

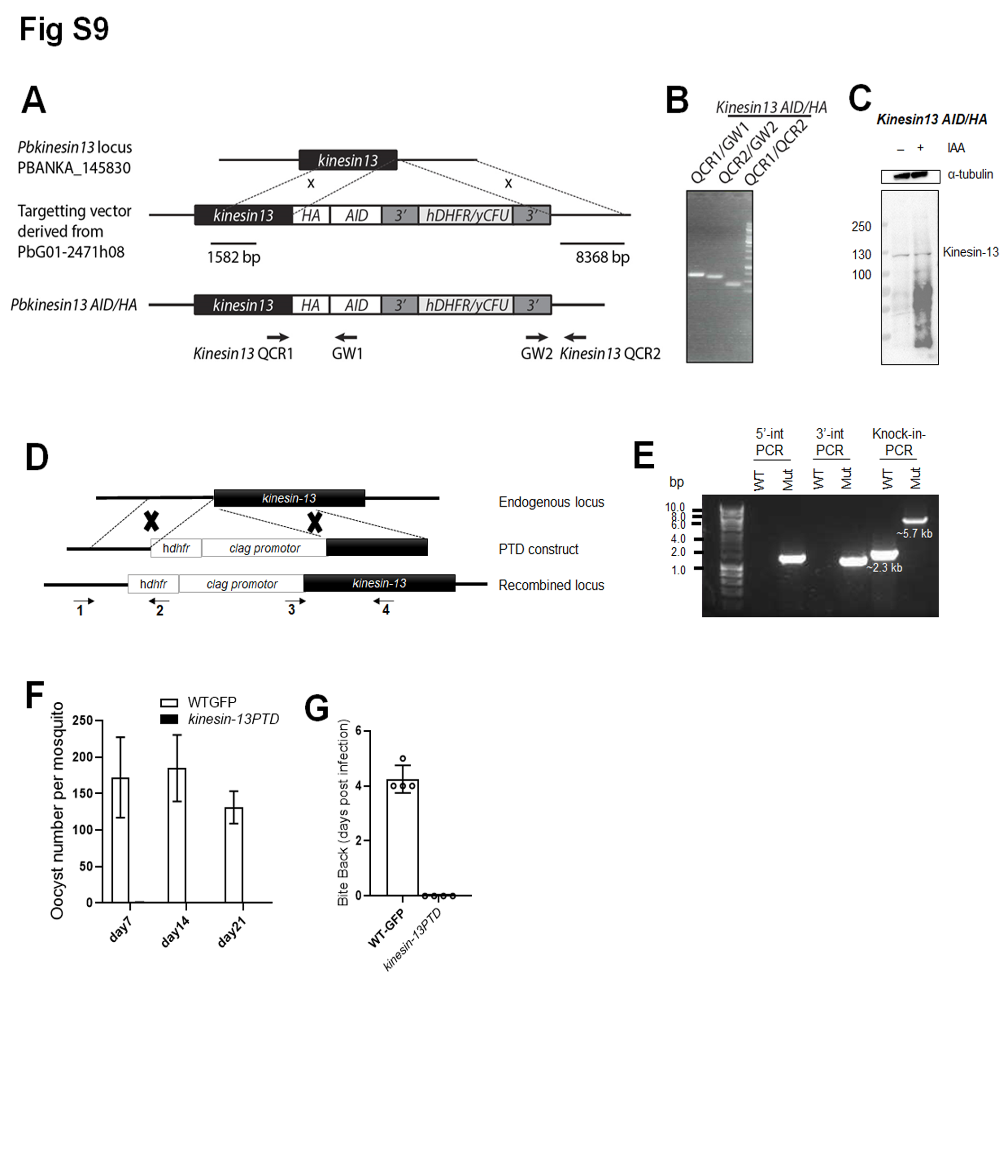

Supplement: S9 Fig — (A) Schematic representation of AID strategy to generate kinesin-13AID/HA parasites. (B) Integration PCR of the kinesin-13AID/HA construct in the kinesin-13 locus. Oligonucleotides used for PCR genotyping are indicated and agarose gels for corresponding PCR products from genotyping reactions are shown. (C) Kinesin-13AID/HA protein expression level as measured by western blotting upon addition of auxin to mature purified gametocytes; α-tubulin serves as a loading control. (D) Schematic representation of the promoter swap strategy (kinesin-13PTD, placing kinesin-13 under the control of the clag promoter) by double homologous recombination. Arrows 1 and 2 indicate the primer positions used to confirm 5′ integration, and arrows 3 and 4 indicate the primers used for 3′ integration. (E) Integration PCR of the promotor swap construct into the kinesin-13 locus. Primer 1 (5′-IntPTD50) with primer 2 (5′-IntPTD) were used to determine successful integration of the selectable marker. Primer 3 (3′-intClag) and primer 4 (3′-IntPTD50) were used to determine the successful integration of the clag promoter. Primer 1 (5′-IntPTD50) and primer 4 (3′-IntPTD50) were used to show complete knock-in of the construct and the absence of a band at 2.3 kb (endogenous) expected if no integration occurred. (F) Oocysts at days 7, 14, and 21 post-infection. n = 3 independent experiments with a minimum of 8 mosquito guts. Error bar, ±SEM. (G) Bite back experiments reveal no transmission of kinesin-13PTD and successful transmission of WTGFP parasites from mosquito to mouse. Mean ± SEM. n = 3 independent experiments. Underlying data are provided in the Supporting information as S5 Data. AID, auxin-inducible degron. (TIF) [file pbio.3001704.s009.tif]
