# Supplementary material for: Genome-wide functional analysis reveals key roles for kinesins in the mammalian and mosquito stages of the malaria parasite life cycle
Source: PLoS Biol. 2022 Jul 28;20(7):e3001704. doi: 10.1371/journal.pbio.3001704 (PMC9333250; doi:10.1371/journal.pbio.3001704)

S1_Raw_Images supporting gels and blots

DNA gels used in Fig S1B.

Gels showing integration PCR products for kinesin-GFP tag lines. Gene names are indicated on the top of box that has been used to show the bands of correct integration, and all unboxed lanes are unrelated to this report.

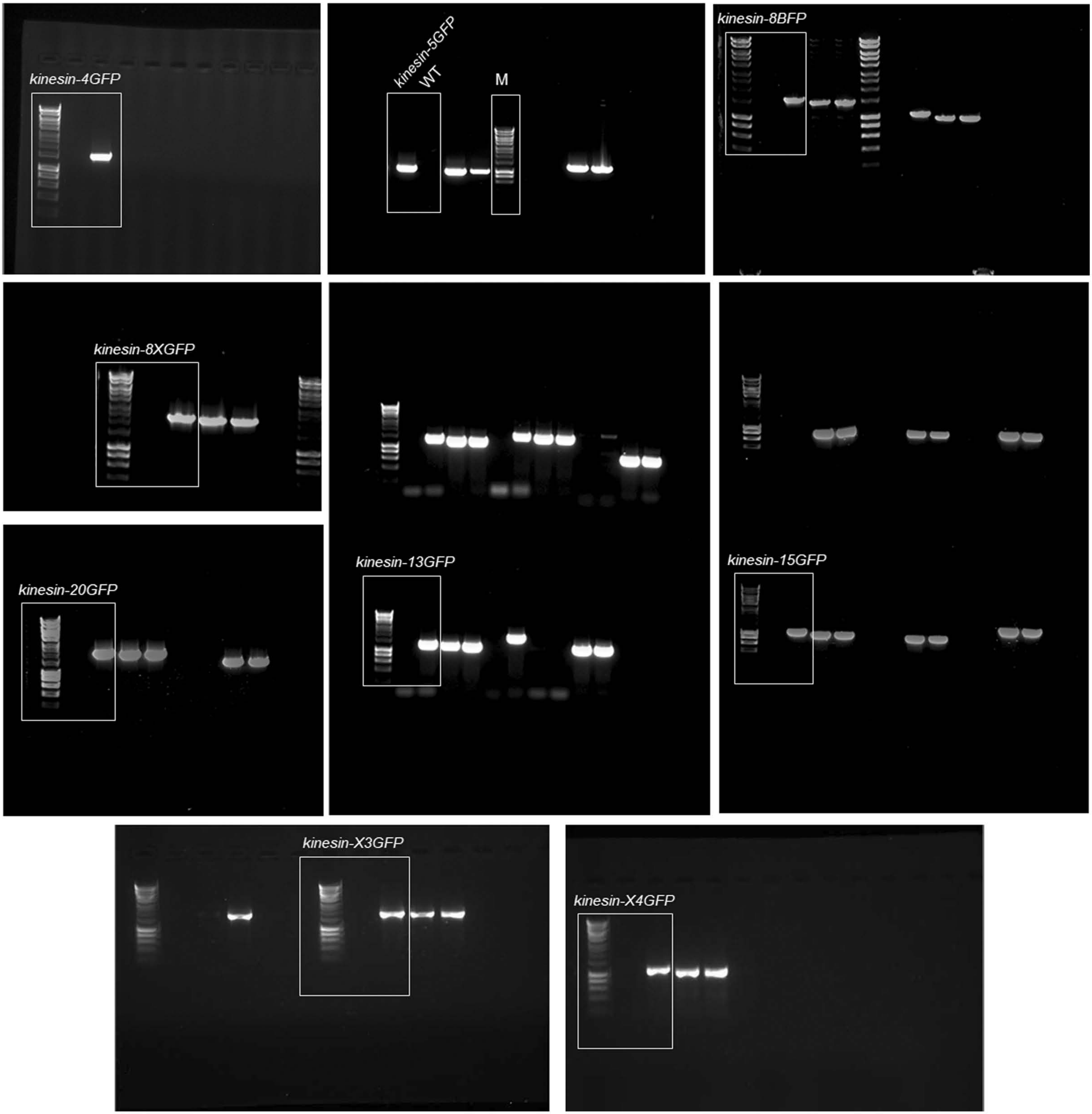

DNA gels used in Fig S4B.

Gels showing integration PCR products for kinesin-knockout lines. Gene names are indicated on the top of box that has been used to show the bands of correct integration, and all unboxed lanes are unrelated to this report.

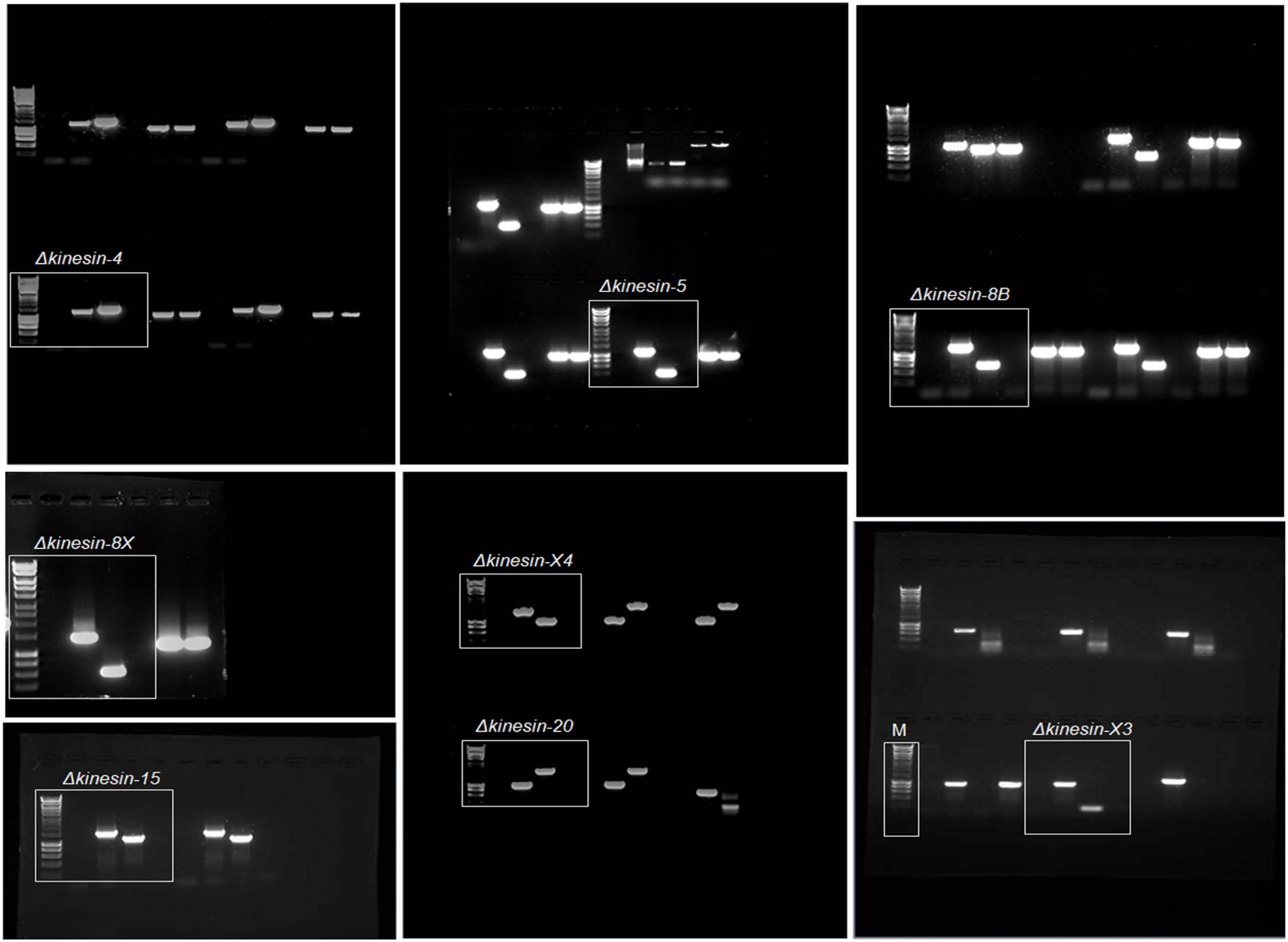

DNA gels used in Fig S9BE.

Gels showing integration PCR products for kinesin-knockdown (AID and PTD) parasite lines. Gene names are indicated on the top of box that has been used to show the bands of correct integration, and all unboxed lanes are unrelated to this report.

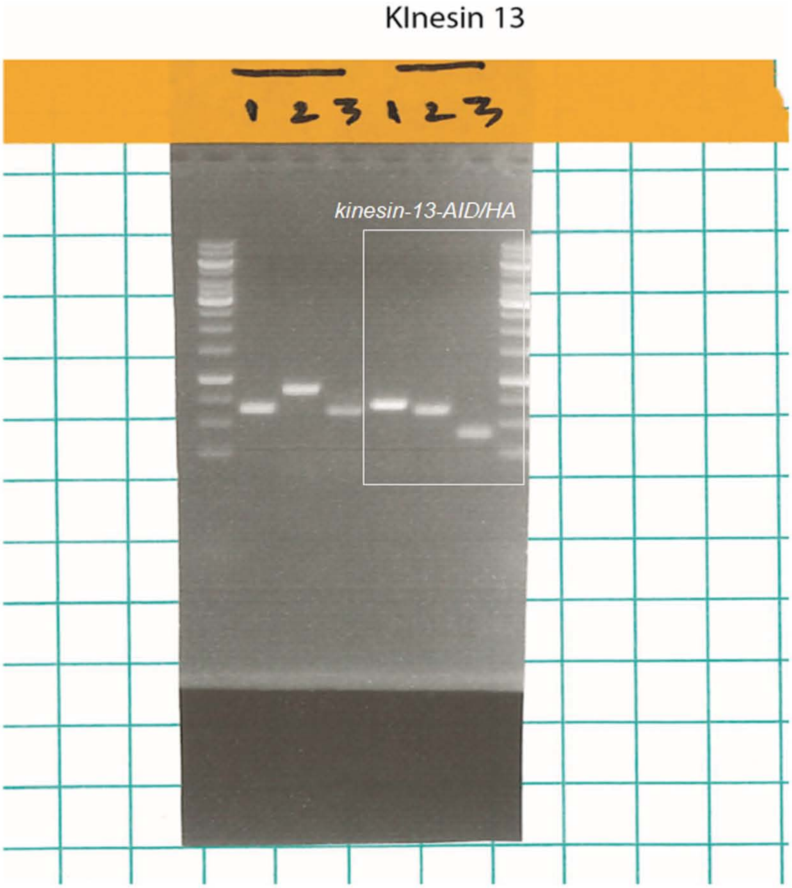

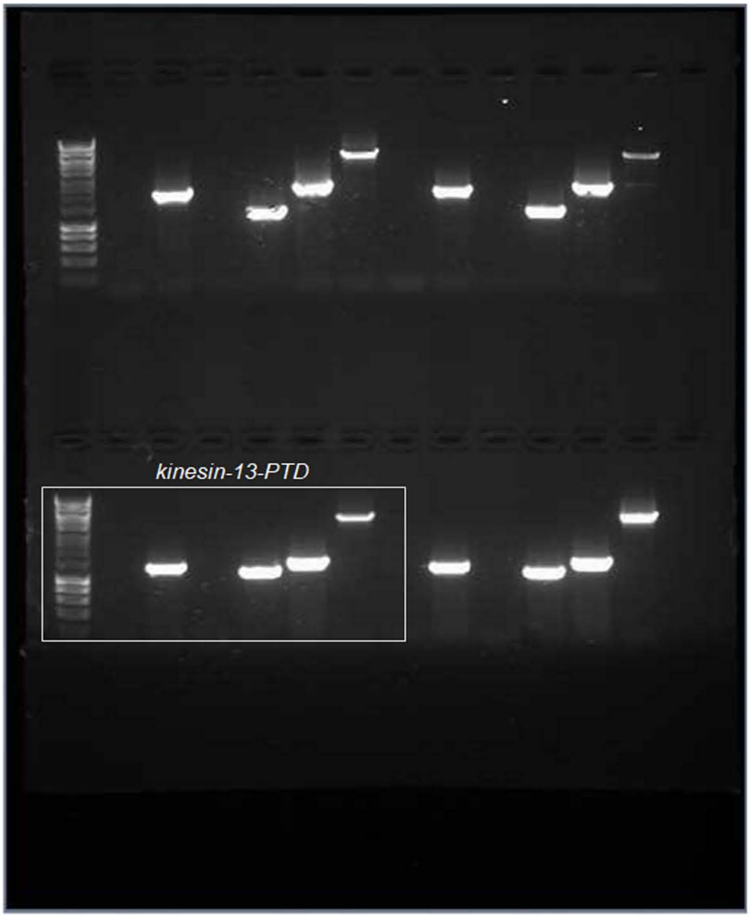

Western blot images used for Fig S9C

Western blots showing the expression of kinesin-13 and α-tubulin in the gametocyte lysate of *kinesin-13-AID/HA* parasite before and after addition of IAA.

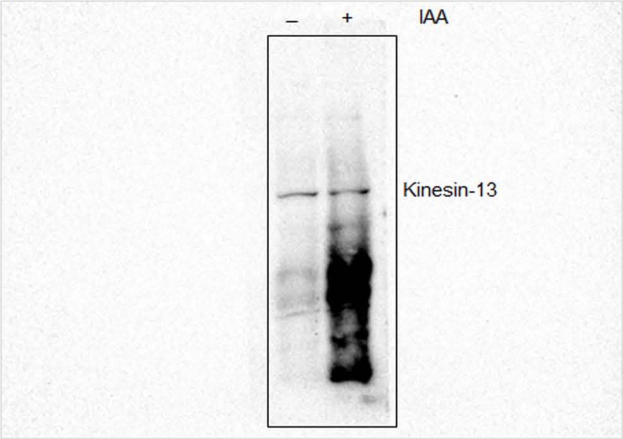

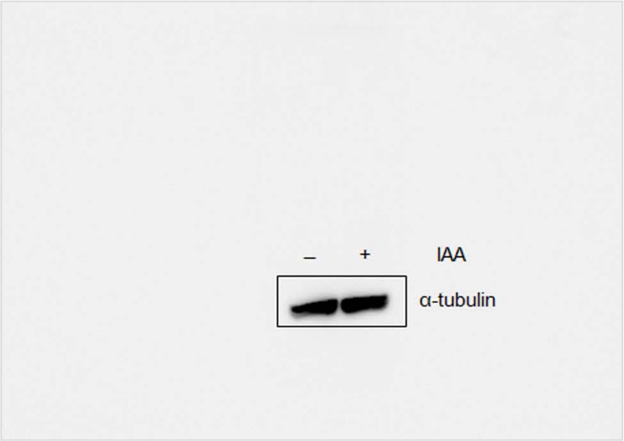

Supplement: S1 Raw Images — (PDF) [file pbio.3001704.s021.pdf]
