# Supplementary material for: Genome-wide functional analysis reveals key roles for kinesins in the mammalian and mosquito stages of the malaria parasite life cycle
Source: PLoS Biol. 2022 Jul 28;20(7):e3001704. doi: 10.1371/journal.pbio.3001704 (PMC9333250; doi:10.1371/journal.pbio.3001704)
Supplement: Attachment — Submitted filename: 121238_1_rebuttal_2314540_r8r5hj.docx [file pbio.3001704.s022.docx]

**Manuscript number:** RC- 2021-01102

**Corresponding author(s):** Rita Tewari; Mohammad Zeeshan

## 1. General Statements [optional]

*We wish to thank the reviewers and the Editor for their constructive comments and valuable suggestions to improve our manuscript. We have addressed as far as possible all comments and concerns and we hope that this revised manuscript, with additional new data, will be acceptable for publication. Please find below detailed responses (in italicised red text) to all specific points raised by the reviewers.*

## 2. Point-by-point description of the revisions

*This section is mandatory.* *Please insert a point-by-point reply describing the revisions that were already carried out and included in the transferred manuscript.*

Reviewer #1 (Evidence, reproducibility and clarity (Required)):

The study led by Dr. Zeeshan analyzes nine mouse Plasmodium parasites kinesin by determining their expression pattern and subcellular location in various stages of the parasites in the mammalian and mosquito host. The genetic and phenotypic analyses of all nine kinesins indicate that most are critical for parasite development in the mosquito host, except for Kinesin 13 being the only kinesin essential during the intraerythrocytic development cycle. The authors presented an in-depth analysis on kinesin 13 and 20, using an impressive pallet of molecular techniques such as promotor swapping, chromatin immunoprecipitation, and global transcriptomic analysis using RNAseq, as well as numerous microscopy techniques such as live fluorescence imaging, expansion microscopy, and electron microscopy. This comprehensive study provides an outstanding amount of data on Kinesins in Plasmodium parasites that would be best showcased with a rethinking of the manuscript structure and a more insightful discussion section that directed most of my comments in the review the manuscript. I believe no additional experiments are needed assuming that the authors will link Kinesin 13 and or 20 to the IMC formation in future work.

*Authors’ response: We are pleased that the reviewer likes this comprehensive study and believes that no additional data are required. We have now reorganised the manuscript with more focus on kinesin 13 and kinesin 20.*

**Major Comments:**

•The current manuscript shows the " Location and function of Plasmodium kinesins" as the title suggests; however, I strongly recommend the authors consider alternative storytelling focusing on Kinesin 13 and 20.

*Authors’ response: We thank the reviewer for this recommendation. We have changed both the organisation of the text and the title of the manuscript to focus on kinesin-13 and -20. The new title of the manuscript is “Key roles for kinesin-13 and kinesin-20 in malaria parasite proliferation, polarity and transmission revealed by genome-wide functional analysis”.*

The author provides in-depth phenotypical analysis resulting in the most innovating and exciting data. In addition, the discussion section from lines 592 to 634 was fascinating compared to the following section (see details comments for Discussion section below).
*Authors’ response: We are very pleased that the reviewer considers the phenotypic analysis to be the most innovative and exciting data, and that they appreciated the discussion section of lines 592 to 634.*

•The following significant comments are related to figures where I believe a restructuration is most needed to bring clarity to the paper.”

•Figure 1. I suggest the authors move Figure 4A to figure 1; Figure1C should move to supplementary information

*Authors’ response: As suggested by reviewer, old Figure 4A is now moved to Figure 1(as Figure 1D) and old Figure 1C is moved to supplementary information (as S3 Fig)*

except for Kinesin 13 and 20 data to center the paper's focus on these two proteins

*Authors’ response: The kinesin-13 and -20 data are now given prominence, as Figures 3 and 4 (kinesin-20) and Figures 5, 6 and 7 (kinesin-13).*

I would also present the kinesin data in the current Figure4A not by numeric order but by biological relevance. All the "normal" together and so on

*Authors’ response: We thank the reviewer for this suggestion; in Figure 1D (previously 4A) the data are now presented in the order of biological relevance*.

•Figure 2: Kinesin 5 and 8X have the same results. I suggest the authors present only one in the same manuscript and place the other one in Supplementary information.

*Authors’ response: kinesin-5 data are now part of S6 Fig in supplementary information and only kinesin-8X data are retained as part of Figure 2*.

I would recommend adding the little schematic used in Figure1C to help the reader quickly identify the parasite stages presented in the figures

*Authors’ response: A schematic is included in Figure 2C for clarification, as suggested.*

•Figure4: Panels B to E should be a supplementary information

*Authors’ response: These panels have now been moved to supplementary information as S5 Fig.*

•Figure 5: Panels H to J should be supplementary information

*Authors’ response: Panels H to J have been moved to supplementary information as part of S8 Fig.*

and I strongly recommend the authors to present data by stages; therefore, I would remove panels F and G and replace them with Figure 6A, the expansion microscopy represents the data in Figure 4B, C, D, and E beautifully

*Authors’ response: we thank the reviewer for this suggestion; expansion microscopy data are now incorporated into the new Figure 3, and the old panels F and G are now part of S8 Fig in supplementary information.*

•Figure 6B: It is challenging to identify the layout between WT and delta-kinesin 20. All annotations on the EM data cover the data itself. I recommend drawing a representative schematic to guide the reader for identification of ultrastructure

*Authors’ response: we have now included a schematic diagram as Figure 4B, to highlight the key ultrastructural features and facilitate their identification by the reader.*

•Figure 8: Panel C and D should be supplementary information and replaced by the accurate colocalization data of Kinesin 13 presented in Supp figure 5

*Authors’ response: the kinesin-13 colocalization data are now in Figure 5, and the previous Figure 8 Panels C and D have been moved to supplementary information.*

In addition, comment line 442 is also actual for the ookinete. The true colocalization is with tubulin in male gamete and gametocytes in figure 5A/B

*Authors’ response: We agree with the reviewer; the colocalization data with tubulin in male gamete and gametocytes are now presented as Figure 6A and B.*

•Figure 9: Panel F to J go to supplementary information and replace with the data in figure 10

*Authors’ response: We understand the reviewer’s concerns, however we would like to include these data in a main figure because they provides important information on the differential regulation of transcripts involved in axoneme biogenesis and chromosome dynamics.*

•Figure 10: Could be a great abstract figure in the current state. As a model figure, I would recommend incorporating more details

*Authors’ response: We have removed this figure (and therefore there are now seven rather than ten figures in the revised manuscript). We would, of course, be happy to use it as part of an abstract if required.*

**Minor Comments:**

I will address my following minor comment by Line number rather than section:

Figure 1C: It is unclear if the black square is an actual picture or a black square. I would suggest the authors present the absence of data by a white square or a bar

*Authors’ response: For this figure (now S3 Fig, previously Figure 1C), we have added the scale bars on the dark squares to indicate that these are actual pictures that show the absence of signal.*

Line 96: " a final synchronized round of S-phase" The classical mitotic terminology is poorly used in the field of Plasmodium mitosis due to the absence of canonical cell cycle checkpoint. I would recommend the authors rephrase as " a final synchronized round of DNA replication."

*Authors’ response: We thank the reviewer for this suggestion. We have now deleted this sentence as part of an effort to make the introduction more concise.*

Line 149-151: Could the authors indicate what stage of the life cycle the work was done?

*Authors’ response: We now indicate the stages in line number 127.*

Line 161: Missing space between the word "parasite and cell"

*Authors’ response: We have deleted this sentence while revising the introduction to make it more concise.*

Line 163: " These findings will inform a strategy ..." Could the authors explain in greater detail how the study is informative for targeting MT motors for therapeutic. I would argue with the authors that it is an overstatement since the paper did not provide structural data on kinesin as a foundation for drug discovery.

*Authors’ response: The sentence is now modified to remove this overstatement, in line number 134-136.*

Line 368: What was the reasoning for examining whether other kinesin genes' expression is misregulated in delta Kinesin 20?

*Authors’ response: The main reason was the expression of other kinesins expressed in the cytoplasmic compartment of ookinete stages, such as kinesin-X3 and kinesin-13; and kinesin-13 that has a key role in MT organisation during ookinete development. Therefore, we expected that the expression of other kinesins including kinesin-13 may be coordinated with that of kinesin-20 and modulated in the kinesin-20 knockout. We have added a sentence for clarification, lines 332-336.*

Line 515: Could the authors define what is a nuclear pole?

*Authors’ response*: *Nuclear pole is a synonym for spindle pole, which is in general usage with reference to electron micrographs. It serves as a microtubule organising centre (MTOC) for mitotic spindles.*

Line: 576 - 579: The authors mention the absence of the IFT component for flagellum assembly due to the assembly of the axoneme in the cytoplasm. It is known that kinesin-2 is required for the anterograde transport in organism building cilia and flagella using IFT. In the current study, kinesin 2 is not part of the nine kinesins; therefore, it is unclear why the authors made these comments and did not reflect on them. I would suggest removing it or comment it.

*Authors’ response: it is well-established that axoneme assembly in Plasmodium gametocytes occurs in the cytoplasm, which does not require IFT, and the absence of a kinesin-2 gene is consistent with that process. In contrast, the location of kinesin-8B, kinesin-X4 and kinesin-13 suggests that they are involved in this non-canonical axoneme assembly. For clarification, we have added a sentence at line numbers 521-522.*

Line 546-560: this entire section of discussion would be best in a review paper. It is a well-written summary of the current literature with no discussion related to the data on the present study; therefore, I suggest the authors remove it from the discussion.

*Authors’ response: This section is now largely removed from the discussion except for a few relevant sentences at lines 509-515.*

Line 561 – 571: Great summary of the Kinesin-13 work without discussion.

*Authors’ response: This part (now at line numbers 595-602) has now been modified so that it is more relevant to the discussion.*

Line 572: What do the authors mean by " these findings"?

*Authors’ response: We have explained the meaning of “these findings” (line number 603).*

Line 573 – 589 (assuming 673-689): The authors miss the opportunity to elaborate on how the depletion of kinesin protein could impact the global transcriptome. Are we looking at downstream effects? I strongly recommend the authors resolve the lack of discussion related to the RNAseq data in the study.

*Authors’ response: we have now improved the discussion of the transcriptome data (line numbers 610-613).*

Reviewer #1 (Significance (Required)):

This study is a tremendous amount of work done rigorously and will advance our knowledge in the biology of Plasmodium parasites. We are in urgent need to develop innovative ways to block the replication and transmission of Plasmodium spp. and it can happen only through advancing our knowledge in the basic biology of the parasite.

*Authors’ response: We thank the reviewer for their positive and encouraging comments.*


Reviewer #2 (Evidence, reproducibility and clarity (Required)):

**Summary:**
In this study, Zeeshan et al used live-cell imaging, ultrastructure expansion microscopy, and electron microscopy, gene deletion, genetic knockdown, RNA-seq, ChIP-seq analyses, and matrigel substrate to examine the subcellular localization and the function of Plasmodium kinesins throughout the P. berghei life cycle. They find that Kinesin-13 is the only kinesin essential for both asexual blood stages and sexual stages.

This manuscript represents a lot of work by the authors. The data appear rigorous and well-executed. The data are clearly presented and the writing is clear. I have only minor comments that may improve the reader's comprehension.

*Authors’ response: We thanks the reviewer for their positive appreciation of our work.*

**Major comments:**

Figure 2C:

The ChIP-seq experiments examined the kinesin-5 and -8x binding site at the chromosome at 6 mpa. Did the authors do any tests at other time points post-activation?

*Authors’ response: We sampled only at 6 mpa because at this time point the expression of kinesin-5 and -8X is high, facilitating the ChIP-seq analysis using anti-GFP antibodies. We now include additional ChIP-seq data in S6 Fig.*

Figure 4:

The authors conclude that kinesin-x3 and kinesin-x4 are non-essential for the P.berghei life cycle. Does deletion of kinesin-x4 affect the length of the flagella?

*Authors’ response: We observed no obvious change in the length of flagella after these deletions.*

Oocyte size: To the non-specialist, it is difficult to reconcile the images in panel E with the conclusions in panel A. Based on the images, it looks like only knocking out of kinesin-8x seems to affect oocyst size. Can the authors clarify and provide graphs of the quantification of oocyte size?

*Authors’ response: We agree with reviewer that only the knockout of kinesin-8X affects oocyst size. Similar data, obtained using live cell fluorescence imaging and electron microscopy, have been described and discussed earlier in Zeeshan et al, 2019 PLOS Pathogens (PMID: 31600347).*

**Minor comments:**

line 190: typo, kinesin-x4

*Authors’ response: This typo has now been corrected (line 162).*

Figure 3: what do the arrows mean?

*Authors’ response: Arrows indicate the pellicle and axonemes that are mentioned in the figure legend (current Figures 2A and B).*

Figure 4F:
 the
1. Typo, scale bar, um.

*Authors’ response: We have corrected this (please see the legend for current S5D Fig).*

2. Does deletion of kinesin-5 show a significant difference?

*Authors’ response: Yes, the number of infective sporozoites in salivary glands is significantly reduced following kinesin-5 deletion (as published previously in the manuscript of Zeeshan et al 2020 [PMID: 33154955]).*


Reviewer #2 (Significance (Required)):

The study provides comprehensive information on the diverse subcellular location and functions of P. berghei kinesins throughout the P. berghei life cycle. That is useful to exploit the therapeutic targets against malaria.

The main findings are that kinesin-13 genetic knockdown affected MT dynamics during spindle formation and axoneme assembly in male gametocytes and subpellicular MT organization in ookinetes. In addition, Kinesin-13 shows different binding to kinetochores during the gametogenesis and ookinete development, suggesting other proteins may regulate kinesin-13 binding to kinetochores at various stages. The underlying mechanism will help to better understand the role of kinesin-13 in the parasite life cycle.


Reviewer #3 (Evidence, reproducibility and clarity (Required)):

In this manuscript the authors show a huge ambition to catalog biological functions of Plasmodium kinesins. This was done by generating transgenic cell lines where kinesins were deleted and/or tagged with GFP that served as a tool to gather as much biological information on each kinesin isoform. On one side I find this manuscript highly impressive in terms of the amounts of data and information. In particular, the cell biology and microscopy results are of high quality and certainly provide useful information to the research community. I am fairly convinced that most results genuinely represents the individual biological aspects of the kinesins in the best possible way. Unfortunately, I have major reservations about the presentation of these results in the compiled manuscript. In my view the authors were overambitious about the volume and diversity of data that they wished to present, which opened a lot of questions about the depth and quality of each of the experimental effort. There is 10 figures which is highly nonstandard for a scientific publication to start with and yet there is, in my view, major gaps in some results descriptions, data presentations e.t.c. Perhaps, because of this huge ambition the data are presented in a highly superficial manner often lacking negative and positive controls. Unfortunately that creates many doubts about the overall quality of the results and as such the interpretations. In my view the authors might be well advised to separate this large body of work into several publications each focusing on more tangible biological problem in the more in-depth manner. This would give the reader (me) better confidence about the validity of the statements made in this manuscript.

I can give few examples of such discrepancies but cannot account for all.

1.The authors created GFP-tagged transgenic cell lines for each of the 9 kinesins and generated life cell images for each of the line across multiple stages of the entire plasmodium life cycle. This is an impressive amount of work and data. It is certainly useful to see that in life cell imaging the different kinesins isoforms can be detected in different sets of developmental stages some diffused in the cytoplasm and some associated with the nucleus. Even though these results are impressive, there are based solely on life cell imaging that rely on a certain level of detection limit and GFP visibility. One can imagine that a kinesin may still be expressed in a developmental stage and not detected by life cell imaging.

*Authors’ response: We agree with the reviewer that live cell imaging has a detection limit for the signal and we cannot rule out the possibility of expression below this limit. We also used immunofluorescence assays (IFAs) to confirm the presence or absence of the proteins at least in the asexual blood- and gametocyte stages. However, our focus was to examine expression by live cell imaging during the transmission stages and hence only those data were given in the manuscript.*

I believe that some other detection methods such a western blot, immunoprecipitation e.t.c. should be provided to truly demonstrate that an individual isoform of a kinesin IS of ISNOT expressed. Without that the Figure 1B is overstated.

*Authors’ response: Some western blots to confirm expression of the intact kinesin-GFP fusion protein have been published previously: for kinesin-8X (PMID: 31600347), kinesin-5 (PMID: 33154955), and kinesin-8B (PMID: 31600347). We now provide immunoprecipitation data for six kinesin-GFP fusion proteins, performed using GFP-trap antibody and with identification by mass spectrometry. These results (S2 Fig) clearly show the presence of the respective kinesins fused to GFP in the immunoprecipitates (S2 fig).*

Moreover, the authors claim that the punctuate signal in the nucleus corresponds to spindle. I do not see any supporting evidence for this in this figure.

*Authors’ response: We have previously provided IFA data using anti-tubulin antibodies (for detection of spindle MT) that clearly show co-localization with nuclear kinesins (kinesin-5 and kinesin-8X). For more detailed information please see Zeeshan et al., 2019, PLOS Pathogen (kinesin-8X; PMID: 31600347) and Zeeshan et al., 2020 Front. Cell. Infect. Microbiol (kinesin-5; PMID: 33154955).*

2.For the analysis of kinesin 5 and 8x the authors note two types of experiments. First they created a "cross" between the two cells lines. Second, the authors carry out ChIP-Seq to show that the proteins localize to the centromere. This could be an impressive result unfortunately there is very little if any information about it. Genetic crosses in Plasmodium are not standard techniques that one can assume works all the time. I believe there should be more evidence that the presented images come from a true genetic cross.

*Authors’ response: We now provide images obtained using both single and dual fluorescence in in the same panel, which show the signal of individual kinesins in different cells as well as both signals in one cell (please see S6A Fig). The two lines, one expressing a GFP-tagged protein and the other an mCherry-tagged protein are crossed by feeding gametocytes together to the mosquito where fertilization and genetic recombination takes place. This genetic cross follows Mendelian rules, producing parental single and two recombinant lines (1:2:1 ratio). The lines are not pure clones but contain parasites that express either both or single fluorescence signals.*

The least the authors could show that the florescence signal for both channels come from genuine integrations of the GFP proteins to their target kinesins by PCR or genome sequencing.

*Authors’ response: We have also confirmed the presence of genes for each tagged protein by integration PCR in these crossed lines, and by live cell imaging as shown in S6A Fig.*

Similarly for the chip-seq, there is a need to provide much detailed information about the entire results with a particular clarity about the position of the peaks in respect to projected centromeres. In addition the ChIP-Seq analysis should be supported by data along with positive and negative controls to truly show the kinesins associations with the centromeres.

*Authors’ response: We provide the positive and negative controls for the ChIP-seq data (please see S7 Fig). The raw data and further details are deposited in the NCBI Sequence Read Archive with accession number: PRJNA731497.*

3.In the middle part, the author present rater impressive analyses of several kinesin deletion trains and their effect on the development of the mosquito stages. In particular, they demonstrate the effect of kinesin 20 on ookinete development. Yet in the next paragraph they present RNA seq analysis of the kinesin 20 deletion on gametocyte induction, in which kinesis 20 should not have any effect; judging from the presented phenotypic assay. This experiment seems out of context as it is unclear why this assay was done and what is the outcome. The authors identified a small group of differentially expressed genes seemingly unrelated to neither kinesin function nor gametocyte induction. This experiment does not make sense to me in the context of the rest of the paper.

*Authors’ response: We agree with the reviewer about the effect of kinesin-20 deletion on ookinete development and the RNAseq analysis of the gametocyte stage.* *This is because kinesin-20 expression starts in female gametocytes and continues into later stages including ookinetes. We know that in Plasmodium there is translational repression in female gametocytes, which de-repress only in the zygote after fertilisation, leading to translation of many proteins in the zygote. We wished to see whether there was a role of kinesin-20 in translational de-repression. Our transcriptomic data showed no role of kinesin-20 in this process. We have added a sentence for clarification in lines 338 -342.*

Reviewer #3 (Significance (Required)):

As mentioned above, these three examples represent some of the discrepancies not necessarily about the data quality and fidelity but rather a confusing character of the entire study. From this perspective I have two types of problems with this manuscript. First, while reading this manuscript, lacking key controls and detailed description of some of the analyses, made me loose interest as well as confidence in other parts of the studies which may or may not be solid. Second, I struggle to see the key purpose of the presentation. Instead the manuscript seems to be a compilation of very diverse data some of which are interesting but other out of context, confusing and not connected to the rest of the study.

*Authors’ response: We are sorry for the confusion, but we have now streamlined the data presented, to focus in the manuscript mainly on two kinesins, kinesin-13 and kinesin-20. We provide the relevant controls and a detailed description of the analyses. All experiments were repeated at least three times, and, where appropriate, figure legends provide information on the number of samples and repeats. We hope the reviewer finds the manuscript clearer now.*

Overall I wish to reiterate that I believe that there are a lot of very good experimental results in this study but unfortunately many of these get lost in the overall presentation that is often superficial or out of context. My general impression is that the authors are trying to show too much, "too fast" and as such many of the presented results remain questionable. The author are likely able to correct all these discrepancies but this might not be possible to do in ne manuscript.

*Authors’ response: We are thankful to the reviewer for finding a lot of very good experimental data and hope that the revised manuscript, with more focus on two kinesins, will give more confidence in our work to the reviewer.*
