# Supplementary material for: Genome-wide functional analysis reveals key roles for kinesins in the mammalian and mosquito stages of the malaria parasite life cycle
Source: PLoS Biol. 2022 Jul 28;20(7):e3001704. doi: 10.1371/journal.pbio.3001704 (PMC9333250; doi:10.1371/journal.pbio.3001704)
Supplement: Attachment — Submitted filename: Response to Reviewers comments-27-05-22.docx [file pbio.3001704.s024.docx]

**Responses to Reviewers’ comments** (In blue)

We thank the academic editor and reviewer for their positive and constructive comments and their suggestions to improve the manuscript, which we have carefully addressed. We hope that these revisions satisfy all the editor’s and reviewer’s queries and concerns. We provide below our responses (highlighted in blue) to specific points made by the reviewer and editor:

**Reviewer's report**:

Reviewer #1: The authors addressed all comments from the Review Commons published in December 2021.
I  have one last request upon endorsement for publication is to remove the term "endomitotic" in line 87.
Endomitosis means that the number of chromosomes increases without nuclear division, leading to the polyploid stage. In contrast, the Plasmodium parasite replicates by schizogony, where DNA replication is followed by nuclear division (PMID: 35353560). For instance, Sarcocystis replicates by endomitosis, not Plasmodium (suggested references are PMID: 17604449 and 33912479).
Understanding the function of microtubule motors in Plasmodium spp will advance our knowledge of the atypical cell division mode of the malaria parasite and unveil innovative ways to block the parasite replication.

We have removed the term, ‘endomitotic’.

We thank the reviewer for their opinion that this study “will advance our knowledge of the atypical cell division mode of the malaria parasite and unveil innovative ways to block the parasite replication”.

**Comments from the Academic Editor:**

Point 1. Details are missing in the methods for how the authors performed the immunoprecipitation with the GFP-TRAP beads and there is also no mention of what served as the negative control. Moreover, the peptide counts in the negative controls should also be included in S2 Fig so we can see what the background counts are like.

We have added to the methods section a description of the immunoprecipitation and mass spectrometry analyses, which includes mention of the negative controls used. The number of peptides in these controls is described in the S2 Fig legend.

Also regarding GFP expression, Fig 1B and 1C don’t completely talk to one another. Because of the large number of proteins they are looking at, they show 1B for all the various localisations and then then 1C as a summative of what they saw (for which we don't see the raw data). In Fig 1B, The GFP signal either localises to the Spindle Pole or Spindle in male gametocytes and it would be good to know which kinesins these were. There are also no pictures of female gametocytes, male gametes or zygotes in Fig 1 B.

We have modified Fig 1B, adding localisation during other stages to align further with Fig 1C. We have already provided S3 Fig to document localisation at various stages including male gametes. The names of the kinesins are mentioned for individual images.

Whilst it has been previously published that Kinesin 5 and 8X co-localise with anti-tubulin antibodies (to detect spindle), I think it is important to show a couple of co-localisation figures in this manuscript since the focus is on spindles and the reader should not have to go to another publication to see what that labelling looks like.

We provide co-localisation images for kinesin-8X with α-tubulin as a marker for spindle (Fig 2C).

Point 2. Chip-Seq data is presented in S7Fig and includes positive and negative controls as requested. However, information regarding the controls should also be in the methodology section.

We have added information regarding the controls in the methodology section. Please see line numbers 888, 892 and 925.

Point 3. Whilst the authors have now made a change in the text regarding why they undertook RNAseq analysis on the gam stage, they didn't actually point out that their transcriptomic data showed no role for kinesin-20 in translational repression. This should be specified as it is simply inferred but not overly clear. Also line349: comma should be replaced with full stop. This suggests a link…..

Translation repression is mentioned now. The comma has been replaced with a full stop. Please see the line numbers 348 and 355.
